# Extracorporeal Life Support: The Next Step in Moderate to Severe ARDS—A Review and Meta-Analysis of the Literature

**DOI:** 10.1155/2019/1035730

**Published:** 2019-09-29

**Authors:** Diamanto Aretha, Fotini Fligou, Panagiotis Kiekkas, Vasilis Karamouzos, Gregorios Voyagis

**Affiliations:** ^1^Department of Anesthesiology and Intensive Care Medicine, General University Hospital of Patras, School of Medicine, University of Patras, Rion, 26504 Patras, Greece; ^2^Technological Educational Institute of Western Greece, Patras, Greece

## Abstract

Despite the use of lung protective ventilation (LPV) strategies, a severe form of acute respiratory distress syndrome (ARDS) is unfortunately associated with high mortality rates, which sometimes exceed 60%. Recently, major technical improvements have been applied in extracorporeal life support (ECLS) systems, but as these techniques are costly and associated with very serious adverse events, high-quality evidence is needed before these techniques can become the “cornerstone” in the management of moderate to severe ARDS. Unfortunately, evaluation of previous randomized controlled and observational trials revealed major methodological issues. In this review, we focused on the most important clinical trials aiming at a final conclusion about the effectiveness of ECLS in moderate to severe ARDS patients. Totally, 20 published clinical studies were included in this review. Most studies have important limitations with regard to quality and design. In the 20 included studies (2,956 patients), 1,185 patients received ECLS. Of them, 976 patients received extracorporeal membrane oxygenation (ECMO) and 209 patients received extracorporeal carbon dioxide removal (ECCO_2_R). According to our results, ECLS use was not associated with a benefit in mortality rate in patients with ARDS. However, when restricted to higher quality studies, ECMO was associated with a significant benefit in mortality rate. Furthermore, in patients with H1N1, a potential benefit of ECLS in mortality rate was apparent. Until more high-quality data are derived, ECLS should be an option as a salvage therapy in severe hypoxemic ARDS patients.

## 1. Introduction

Despite the use of lung protective ventilation (LPV) strategies (low-pressure, low-volume ventilation techniques) to reduce ventilation-induced lung injury [1], a more severe form of acute respiratory distress syndrome (ARDS) has been associated with high mortality rates, sometimes exceeding 60% [2–4]. For the most refractory forms of respiratory failure, venovenous extracorporeal membrane oxygenation (VV-ECMO) is the preferred configuration for extracorporeal life support (ECLS), while in less severe forms of ARDS, low-flow extracorporeal carbon dioxide removal (ECCO_2_R) could be applied, allowing ultra-LPV with lower airway pressures, tidal volume (*V*_T_), and respiratory rates rather than improving oxygenation [5]. Venoarterial ECMO (VA-ECMO) is usually restricted to patients with left or right ventricle failure. However, recently, major technical improvements have been applied in ECMO systems [6–8], but because these techniques are costly and are associated with very serious adverse events, high-quality evidence, especially from randomized controlled trials (RCTs), is needed before these techniques can become the “cornerstone” in managing moderate to severe ARDS and refractory hypoxemia. Unfortunately, the evaluation of previous RCTs and observational trials has revealed major methodological issues. In this review, we focus on the most important clinical trials to unveil a final conclusion about the effectiveness of ECMO and ECCO_2_R in moderate to severe ARDS patients.

## 2. Methods

### 2.1. Database Search and Study Selection

Two reviewers (DA and VK) systematically and independently searched for clinical studies by using combinations of the following search terms: “extracorporeal life support,” “extracorporeal membrane oxygenation,” “extracorporeal carbon dioxide removal,” “hypoxemia,” “acute respiratory distress syndrome,” “mortality,” and “outcome.” The US National Library of Medicine (PubMed), Web of Science, Cochrane Library, and Excerpta Medical Database (EMBASE) were included in the search, which initially took place in the first week of September 2018 and then was updated with additional information in the second week of November 2018 and in the last week of May 2019. First, studies that were retrieved were screened according to their titles and abstracts. Only studies on humans and that had an English abstract were included for screening. Second, the full text of the selected articles was evaluated to make a final determination for inclusion. Finally, the reference lists of the eligible articles were checked for potentially relevant articles (not included in the first online searches). The full texts of these additional articles were also studied for eligibility and possible inclusion. Any discrepancies between the two reviewers were discussed with a third reviewer (FF) until a consensus had been achieved.

**Figure 1:**
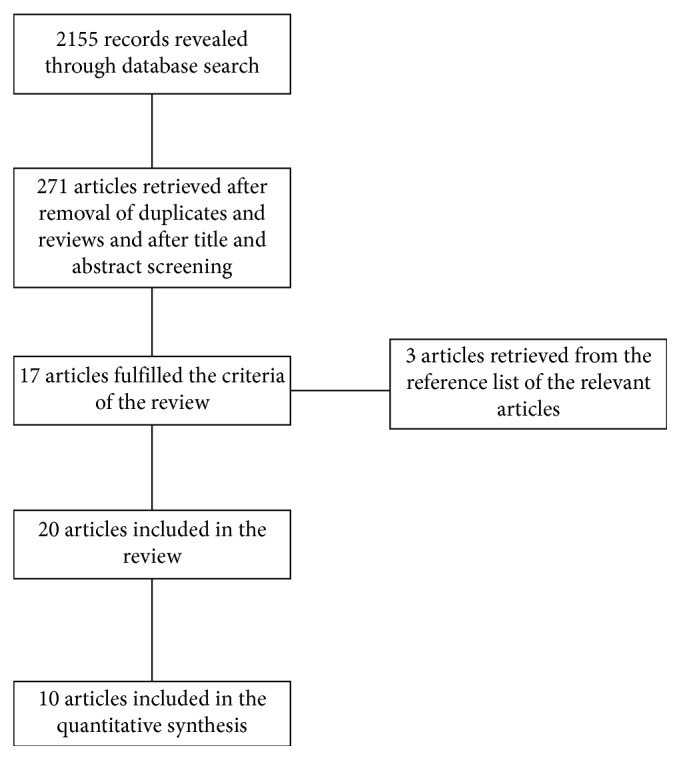
Different phases of information flow of the review.

In total, 20 published clinical studies were included in the current review. The first electronic online database search revealed 2,155 articles for evaluation, of which 271 full-text articles were retrieved for further adjudication and full-text review after the removal of duplicates and reviews and after the title and abstract screening. Finally, 17 articles fulfilled the criteria of the review, while three more articles were retrieved from the reference list of relevant articles, which were then included in the final review as well (totaling 20). Different phases of the information flow of the review are presented in Figure 1.

### 2.2. Outcome, Subgroup Analysis, and Statistics

Our main outcome of interest was hospital mortality, and if this was not provided, then we used ICU or 6-month mortality. To find a possible conclusion, although risky, we decided to pool the results of the eight VV-ECMO trials (two more recent RCTs, two quasi-RCTs, and four prospective observational trials) together [9–16]. We conducted three subgroup analyses restricted to studies with high methodological quality and a low risk of bias: (1) VV-ECMO subgroup analysis (four studies, two RCTs, and two quasi-RCTs), (2) VV-ECMO subgroup analysis in the two most recent RCTs, and (3) ECCO_2_R subgroup analysis (two RCTs). We did not include the RCT of Zapol et al. in any quantitative analysis because this is an old study with major limitations, where only VA-ECMO was used with old technological instruments [17]. Furthermore, the two ECCO_2_R RCTs were quantitatively analyzed separately. We also distinguished ECMO from ECCO_2_R in the presentation of the studies. This distinction could be considered somehow questionable as broad consensus or standardization on this issue does not exist and there is considerable overlap between the two techniques. Nevertheless, although ECMO remains the first choice in patients with refractory hypoxemia, ECCO_2_R is usually used in less severe cases of ARDS with insufficient CO_2_ excretion or uncontrolled hypercapnia in an attempt to facilitate or extend low-volume, low-pressure ventilation. The key difference between the two techniques is blood flow rates. In the ECCO_2_R technique, low blood flow rates (0.4–1 lt/min) are used, with partial respiratory support and without significant effect on blood oxygenation. Instead, in the ECMO technique, blood flows of 3–7 lt/min are used, providing total respiratory support with significant oxygenation and CO_2_ removal. Furthermore, compared to ECMO, ECCO_2_R cannulas are smaller (between 13 and 19 Fr) and conceptually identical to dialysis catheters. Generally, the ECCO_2_R procedure is simpler and with fewer adverse events as well [18–22].

Dichotomous outcomes were reported using odds ratios (ORs) and their 95% confidence intervals (CIs). Heterogeneity among the studies was determined by calculating the *Q* and the *I*^2^ statistic. For the *Q* statistics, a *P* value <0.05 was selected for high heterogeneity, while for the *I*^2^ statistics, heterogeneity was classified as being high when greater than 75%, moderate when between 50% and 74%, and low when less than 25%. In the presence of low heterogeneity (PQ < 0.05, *I*^2^ < 25%), a fixed-effects (FE) model was used, while in the case of moderate or high heterogeneity, a random-effects (RE) model was used. To assess publication bias, funnel plots (treatment difference vs. study precision) and a linear regression analysis (Egger's test) were used. The data analysis was conducted using the Meta-Essentials tool for meta-analysis [23] and SPSS version 22 (IBM SPSS Statistics for Mac, Armonk, NY, USA).

## 3. Trials Evaluating ECMO

Currently, three RCTs are evaluating ECMO. Recently, we read the long-awaited prospective RCT by Combes et al., which evaluated the efficacy of VV-ECMO in patients with severe ARDS (EOLIA trial) [9], but many conclusions of their analysis are problematic. First, according to the study results, the authors concluded that, among the patients with severe ARDS, there was not a statistically significant difference with ECMO use than with a strategy of conventional mechanical ventilation (44 of 124 patients (35%) and 57 of 125 (46%) died in the ECMO and control groups, respectively (relative risk, 0.76; 95% confidence interval (CI), 0.55 to 1.04; *P* = 0.09)). We think that the 11% difference in 60-day mortality between the groups, although not statistically significant, is certainly clinically important. According to the study design, a 20% difference in mortality between the groups, although impressive, was perhaps not realistic. In our opinion, a lower effect size would be more appropriate, but in that case, more patients would be needed in each group. The study is underpowered to detect a 10% difference between groups. Furthermore, considering the 95% CI, which mostly lies below 1, and the fact that very sick patients cross over to ECMO, we have serious clues that a differently powered study would have favoured ECMO. This belief is reinforced not only by the secondary end-point results of the study (treatment failure at 60 days (RR, 0.62; 95% CI, 0.47 to 0.82; *P* < 0.001)) but also by a recently published post hoc Bayesian analysis of the EOLIA study [24]. According to that analysis, the posterior probability of any mortality benefit (relative risk <1) with early ECMO use is high, ranging between 88% and 99%. Furthermore, posterior probabilities for a reduction in mortality with early ECMO use given the EOLIA trial and results from previous studies in severe ARDS patients are very encouraging. Second, a major concern arises from the fact that investigators, even in the conventional mechanical ventilation group, included ECMO as a rescue therapy (ethical issue), making it very difficult to draw definitive conclusions regarding the usefulness of ECMO.

Another prospective RCT—the CESAR trial—was conducted in the UK from 2001 to 2006 (published in 2009) to evaluate VV-ECMO for severe ARDS [10]. In that trial, patients with refractory ARDS were assigned to an expert single center for VV-ECMO consideration, while the patients randomized in the control group were treated at designated centers using LPV. The study enrolled patients from 68 centers and yielded promising results: 6-month mortality or severe disability (both were primary outcomes) was significantly lower in the 90 VV-ECMO group patients (37% vs. 53%, *P*=0.03). The authors concluded that severe hypoxemic ARDS patients who were transferred to a specialized ECMO center showed a significant improvement in survival without severe disability at 6 months. Unfortunately, the study suffered from major methodological issues that limit these final conclusions. First, LPV was not standardized, especially for the control group, while VV-ECMO patients spent significantly more time under LPV. Second, 22 patients randomized to the VV-ECMO group did not receive ECMO at all. Third, five VV-ECMO group patients died during transportation to the ECMO center before receiving ECMO. Finally, for three control group patients, disability information at the 6-month follow-up was missing. The investigators considered an intention-to-treat primary analysis, which was absolutely correct, but if a per-protocol analysis had been used, the final results might have been different.

The procedure was first evaluated in 90 patients suffering from refractory hypoxemia in an RCT conducted in the United States in the 1970s and was sponsored by the National Institutes of Health [17]. In that trial, mortality was more than 90% in both groups, and it was terminated early while ECMO use showed no improvement. Furthermore, the study suffered major limitations. First, the ECMO group of patients received no LPV, resulting in severe complications related to barotrauma. Second, the ECMO mode that was used was only venoatrial and circuits were not heparin coated, resulting in high levels of anticoagulation and a high percentage of patients with severe hemorrhage complications (patients received approximately 4 L blood products per day). Third, ECMO was removed from the patients after 5 days if no improvement was observed, precluding any possible late improvement of these patients.

In 2009, during the influenza A (H1N1) pandemic, patients with severe respiratory failure who received ECMO therapy demonstrated a survival benefit, but the studies they were included in were not randomized. Nevertheless, two quasi-RCTs evaluated ECMO during that period. First, a matched-control observational study with 75 patients from the UK reported an impressive lower mortality rate in ARDS patients suffering from H1N1 influenza A who were treated with VV-ECMO compared with the no ECMO therapy ARDS patients (23.7% vs. 52.5%, *P*=0.006, for individual matching; 24% vs. 46.7%, *P*=0.008, for propensity score matching; and 24% vs. 50.7%, *P*=0.001, for GenMatch matching) [11]. The study used three different forms of case matching from the observational data to minimize confounding in the estimation of therapy effectiveness [25, 26].

Second, in 2013, a new study coming from France—the largest cohort of influenza A- (H1N1-) related ARDS treated with ECMO—failed to demonstrate a clear better outcome in patients treated with ECMO [12]. In the study, 103 ARDS patients who received ECMO within the first week of mechanical ventilation were compared with 157 patients who had severe ARDS but did not receive ECMO. This was also a matched case-control study in which only the unmatched, younger, and severely hypoxemic ECMO-treated patients showed lower mortality rates. Generally using a well-matched subgroup of patients, no differences in mortality existed between ECMO-treated and conventionally treated patients (50% vs. 40%, *P*=0.32). The investigators concluded that ECMO treatment might not be generalizable to all patients with ARDS from various causes. Indeed, the effectiveness and safety of VV-ECMO in patients with ARDS remain an open question. According to the results in ECMO patients, an ultra-LPV strategy may be needed to improve outcomes.

Furthermore, ECMO was evaluated in four prospective observational studies. In 2010, in a prospective observational study of patients treated for confirmed influenza A- (H1N1-) related ARDS, the investigators concluded that ECMO may be an effective salvage treatment, especially for patients presenting with rapid refractory respiratory failure, particularly when presented early on at a specialized reference center [13].

Four years earlier, in 2006 in a prospective observational study, ECMO treatment failed to show any mortality benefit [14]. In that study, mortality in the ECMO-treated patients tended to be higher than that with conservative treatment (46.9% vs. 28.8%, *P*=0.059), but these patients were also significantly sicker.

In 2000, in another prospective uncontrolled observational study, the investigators presented their 10 years of experience working with ECMO [15]. Of 245 patients who suffered severe ARDS, 62 were treated with ECMO. The survival rate in the non-ECMO-treated patients was 61% (not significantly different from that in the ECMO-treated patients).

In 1997, in a prospective uncontrolled observational study, a high survival rate in 122 ARDS patients was achieved by using a clinical algorithm including ECMO [16]. In the non-ECMO patients, the survival rate was 89%, while in the ECMO patients, the survival rate was 55%.

Many retrospective observational studies evaluated ECMO too. In a recently published, multicenter, retrospective unmatched and matched cohort study, VV-ECMO use was compared with conventional mechanical ventilation in severe hypoxemic patients showing lower mortality risk and a longer length of ICU stay [27].

In 2015, in a retrospective score-matched study, patients suffering from ARDS who were treated with ECMO had higher hospital survival rates than patients with a similar disease severity score and at a similar age who were not treated with ECMO [28].

Instead, in 2009, in a retrospective observational study coming from Australia and New Zealand (ANZ), investigators showed that patients who were treated with ECMO had a longer duration of mechanical ventilation and ICU stay and greater ICU mortality [29].

In 2011, the Italian ECMO network experience during the 2009 influenza A (H1N1) pandemic was published [30]. Overall, survival to hospital discharge was 68.3% (41 patients). Interestingly, the rate of ECMO-associated major complications was low, the survival rate was higher in patients who received ECMO earlier, and ECMO allowed for the safe transportation of patients otherwise deemed too sick for a safe transfer.

Finally, in January 2018 in a retrospective observational trial, the 6-month outcomes of immunocompromised severe ARDS patients rescued by ECMO were evaluated [31]. The investigators concluded that a low 6-month survival rate of immunocompromised patients supports the ECMO restriction for patients with a more realistic prognosis, acceptable functional status, and few pre-ECMO mortality risk factors.

## 4. Trials Evaluating ECCO_2_R

Currently, two RCTs are evaluating ECCO_2_R. Low-flow ECCO_2_R, including pressure-controlled inverse ratio ventilation, was used in an RCT in the 1990s, but the trial stopped early after 40 patients had been enrolled [32]. Among the 21 patients that were randomized to the ECCO_2_R group, 30-day survival was 33% vs. 42% in the control mechanical ventilation group (*P*=0.8), while numerous hemorrhagic complications were also present in the ECCO_2_R group, which was the main criticism of the study. The study was terminated after the second interim analysis and after 40 patients had been included. The authors concluded that ECCO_2_R therapy should not be an option in ARDS patients.

The AV-ECCO_2_R method was reevaluated in a prospective RCT with 79 patients (the Xtravent trial) [18]. In the trial, the investigators compared lower tidal volume strategy (3 ml/kg) combined with AV-ECCO_2_R removal versus “conventional” LPV (6 ml/kg, ARDSNet strategy) in severe ARDS [18]. At 60 days, the number of ventilator-free days was not different between the groups (33.2 for the study group vs. 29.2 for the control group, *P*=0.47). The post hoc subgroup analysis, however, revealed that patients with a PaO_2_/FiO_2_ ratio ≤150 mmHg at randomization had significantly more mechanical ventilator-free days at 28 and 60 days and had a more rapid weaning from mechanical ventilation. The mortality rate was low and did not differ between the groups (16.5%). The investigators concluded that AV-ECCO_2_R could be a valuable tool in patients with severe ARDS because it allows for the use of very low tidal volumes. The severe complications occurring during AV-ECCO_2_R use were always a concern, but in the study, only moderate complications occurred in three patients (7.5%), and none of these complications led to permanent impairment.

Recently, ECCO_2_R feasibility and safety were assessed in a single-arm study (the SUPERNOVA study) [33]. In that prospective, multicenter, international, phase 2 study, 95 patients were enrolled, and 78 of them (82%) achieved ultraprotective ventilation by 24 hours. This study showed that ECCO_2_R could be used to minimize respiratory acidosis while applying an ultraprotective ventilatory strategy in patients with moderate ARDS. However, considering the relatively high levels of adverse events, the investigators concluded the need for RCTs to assess if the benefits of the technique outweigh its risks.

Furthermore, ECCO_2_R was evaluated in an uncontrolled study of 43 severe ARDS patients, which was published in 1986 [34]. In the study, lung function was improved in 31 patients (72.8%), and finally, 21 patients (48.8%) survived, while no major technical accidents occurred after more than 8,000 hours of perfusion. The investigators concluded that ECCO_2_R was a safe and valuable tool for severe ARDS treatment.

In 2009, in an LPV clinical trial where the investigators verified whether *V*_T_ lower than 6 ml/kg might enhance lung protection, consequent respiratory acidosis was managed by ECCO_2_R [35]. The authors concluded that *V*_T_ lower than 6 ml/kg enhanced lung protection. Furthermore, respiratory acidosis was safely and effectively managed by ECCO_2_R. In total, 32 ARDS patients were enrolled, while ECCO_2_R was used only in 10 patients.

## 5. Results

### 5.1. Study Characteristics

Most studies have important limitations regarding quality and design, with substantial qualitative heterogeneity among them. In the 20 included studies (2,956 patients), 1,185 patients received ECLS. Of them, 976 patients received ECMO and 209 patients received ECCO_2_R. When ECMO was used, the investigators mainly used VV-ECMO, but in a small number of patients, VA-ECMO was also used. Seven studies (428 ECMO patients) had mainly enrolled patients suffering from H1N1-associated ARDS, while one study (203 patients) enrolled immunocompromised patients. The bias level was estimated using the Cochrane Collaboration risk-of-bias instrument [36]. Generally, there was a significant level of heterogeneity across the clinical trials, which made it risky to pool the data into a meta-analysis. The Cochrane Collaborative Review Groups also concluded this in a recent systematic review [37]. Nevertheless, except for the narrative review of the most important studies, high methodological quality ones were also quantitatively analyzed.

### 5.2. Study Quality Appraisal and Bias Assessment

The RCTs, quasi-RCTs, observational studies, and upcoming RCTs of ECLS are presented in Table 1. Mortality rates in the ECLS group were highly variable in the included studies (range, 24–90%), with the lowest mortality rates reported in the studies published in the most recent years. LPV in both the ECLS and control groups was referred to in six studies [9, 10, 12, 13, 15, 18], while in two studies, LPV was referred to only in the ECLS group [11, 14] and in one study only in the control group [16] (Table 1). A low risk of bias was estimated for the five RCTs (three evaluating ECMO and two evaluating ECCO_2_R) and the two quasi-RCTs [9–12, 17, 18, 32], whereas all the other observational studies had sicker patients in the ECLS group, limiting the comparison of the two different modalities in a similar group of patients. The study distribution was relatively symmetrical on both sides of the mean; thus, concerns for publication bias were not raised, while no significant small study effects were indicated by Egger's test (*P*=0.33).

### 5.3. Quantitative Synthesis of the Study Findings

**Figure 2:**
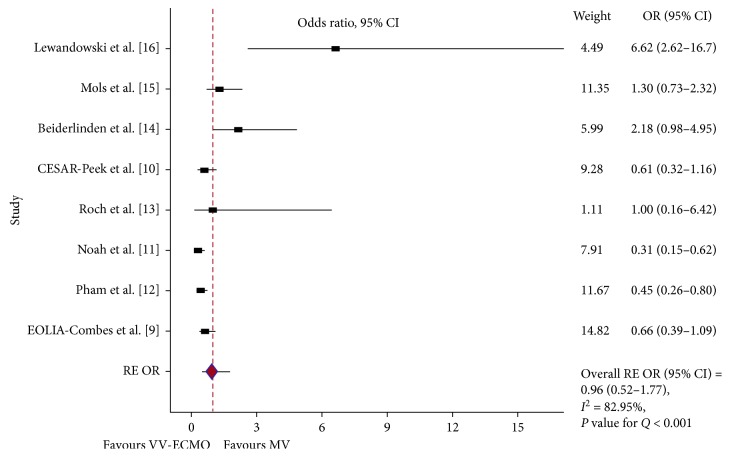
In-hospital mortality: forest plot showing the pooled analysis of eight higher quality studies when the ECLS modality was VV-ECMO. The GenMatch data were used for the Pham and Noah studies, while the per-protocol analysis was used for the Peek study. Using a random-effects (RE) model: odds ratio (OR) = 0.96; 95% confidence interval (CI) = 0.52–1.77; *I*^2^ = 82.95%; *P* value for *Q* (PQ) < 0.001.

**Figure 3:**
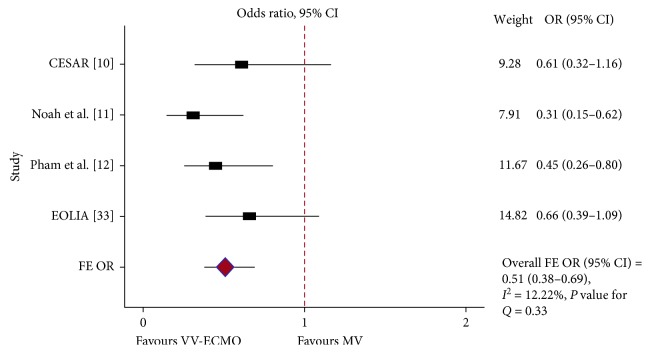
In-hospital mortality: forest plot showing the subgroup pooled analysis of two RCTs and two quasi-RCTs when the ECLS modality was VV-ECMO. The GenMatch data were used for the Pham and Noah studies, while the per-protocol analysis was used for the Peek study. Using a fixed-effects (FE) model: odds ratio (OR) = 0.51; 95% confidence interval (CI) = 0.38–0.69; *I*^2^ = 12.22%; *P* value for *Q* (PQ) = 0.33.

**Figure 4:**
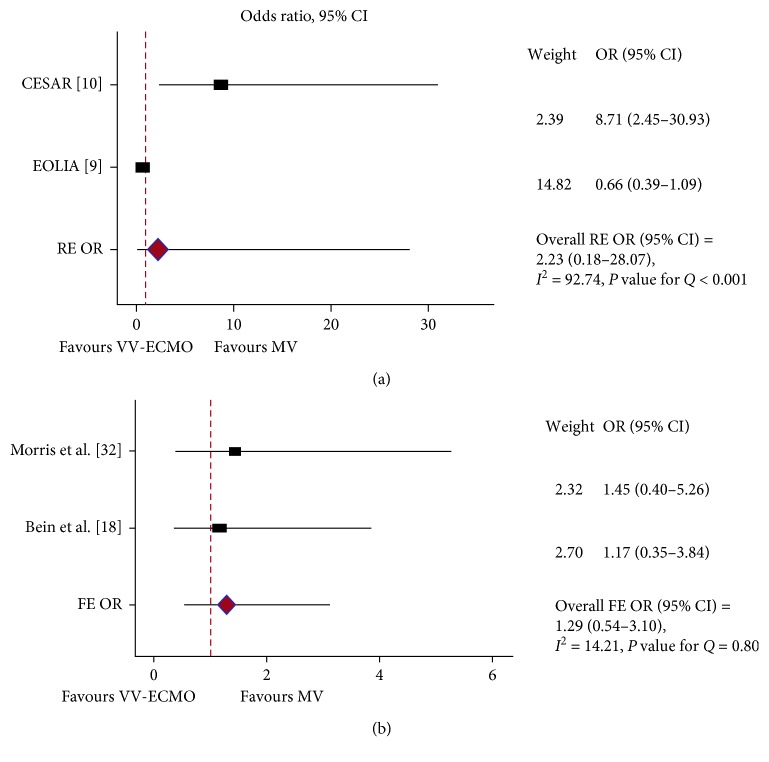
In-hospital mortality: forest plots showing the subgroup pooled analysis of two RCTs when the ECLS modality was VV-ECMO (CESAR and EOLIA) (a) and VV-ECCO_2_R (Morris and Bein) (b). The per-protocol analysis was used for the CESAR study. (a) Using a random-effects (RE) model: odds ratio (OR) = 2.23; 95% confidence interval (CI) = 0.18–28.07; *I*^2^ = 92.74%; *P* value for *Q* (PQ) < 0.001; (b) using a fixed-effects (FE) model: odds ratio (OR) = 1.29; 95% confidence interval (CI) = 0.54–3.10; *I*^2^ = 14.22%; *P* value for *Q* (PQ) = 0.80.

Hospital mortality was reported in 11 studies. Of them, 10 studies included in the pooled results (of 1,497 patients, 1,040 received ECLS). Putting the results of the two RCTs [9, 10], the two quasi-RCTs [11, 12], and the four prospective observational trials [13–16] together, ECMO failed to show any survival benefit in ARDS patients (Figure 2). Because PQ < 0.001 and *I*^2^ = 83%, a RE model was used (RE OR = 0.96, 95% CI = 0.52–1.77). In the subgroup analysis, when restricted to RCTs [9, 10] and quasi-RCTs [11, 12], there was a mortality difference favouring the ECMO group (PQ = 0.33, *I*^2^ = 12.2%, FE OR = 0.51, 95% CI = 0.37–0.70) (Figure 3). Furthermore, pooling the results of the two most important RCTs on this issue together [9, 10], the ECMO procedure does not favour severe ARDS patients (PQ < 0.001, *I*^2^ = 92.7%, RE OR = 2.23, 95% CI = 0.18–28.07) (Figure 4). Finally, the results of the two RCTs evaluating ECCO_2_R [32, 33] showed no survival benefit in the ECCO_2_R group versus the control group (PQ = 0.8, *I*^2^ = 13%, FE OR = 1.29, 95% CI = 0.54–3.10) (Figure 4).

### 5.4. Adverse Events

**Figure 5:**
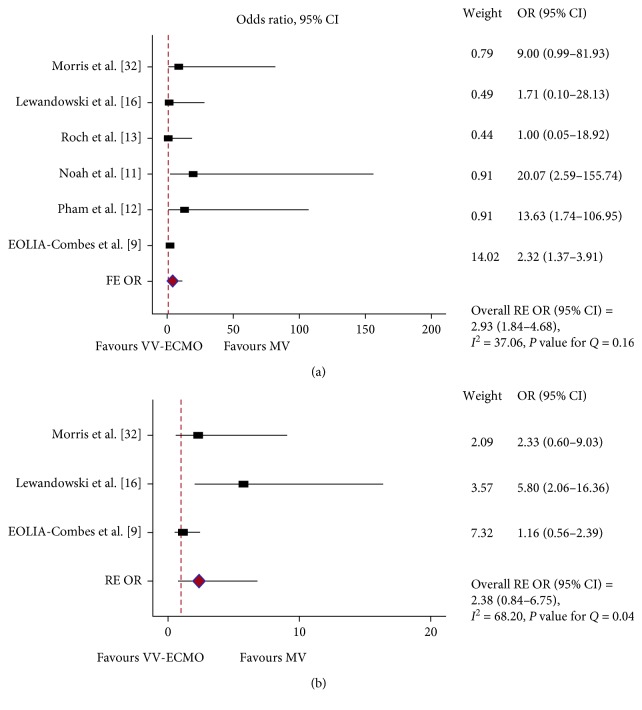
Adverse events: forest plots showing the subgroup pooled analysis of bleeding (a) and barotrauma/pneumothorax (b) for both VV-ECMO and VV-ECCO_2_R. (a) Using a fixed-effects (FE) model: odds ratio (OR) = 2.93; 95% confidence interval (CI) = 1.84–4.68; *I*^2^ = 37.06%; *P* value for *Q* (PQ) = 0.16; (b) using a random-effects (RE) model: odds ratio (OR) = 2.38; 95% confidence interval (CI) = 0.84–6.75; *I*^2^ = 68.20%; *P* value for *Q* (PQ) = 0.04.

With the exception of the EOLIA trial [9], the reporting and definitions of adverse events were usually absent and, if present, heterogeneous among the studies. We analyzed the incidence of bleeding and barotrauma/pneumothorax (Figure 5). Bleeding was more common in the ECLS groups (PQ = 0.16, *I*^2^ = 37%, FE OR = 2.93, 95% CI = 1.84–3.68). Regarding barotrauma/pneumothorax, there were no significant differences between groups when the RE model was considered (PQ = 0.04, *I*^2^ = 68%, RE OR = 2.38, 95% CI = 0.84–6.75). When the FE model was considered, the barotrauma/pneumothorax events were more common in the ECLS group (FE OR = 2, 95% CI = 1.17–3.48). Of note, with the exception of the EOLIA trial, the barotrauma/pneumothorax reports were coming from studies before the institution of LPV modes.

## 6. Discussion

Our review and meta-analysis of 20 studies including 2,956 patients revealed no significant differences in mortality in patients with ARDS treated with ECLS. However, when limited to higher quality studies, ECMO reduced in-hospital mortality when compared with conventional mechanical ventilation techniques. Furthermore, patients with H1N1-associated ARDS showed a significant mortality benefit from ECLS, especially younger ones. Indeed, the potential benefits, such as adequate gas exchange and reduced ventilation-induced lung injury, should be balanced against the risks associated with bleeding, barotrauma, hemolysis, catheter-related infections, thrombosis, air embolism, and so forth. Bleeding was found to be the major adverse event associated with ECLS, while barotrauma/pneumothorax was probably higher in the ECLS groups too. However, the newest generation of devices seems more biocompatible and less stimulatory of the inflammatory and coagulation cascades. Generally, the newest devices are more efficient because they require lower anticoagulant doses and they are associated with fewer hemorrhagic complications [38].

To the best of our knowledge, our review and meta-analysis is the largest cohort of ECLS use in ARDS patients evaluating mortality. The inclusion of RCTs, quasi-RCTs, and observational trials (prospective and retrospective) and the narrative analysis of them together with the meta-analysis of the most high-quality studies allow for a more robust assessment of the potential impact of ECLS on ARDS. In two recently published reviews and meta-analysis studies, the results were similar to those of the current study [39, 40]. Compared with previous studies, our study is heavily influenced by the recently published EOLIA trial [9], which was not included in the previous reviews. The conclusions of the EOLIA trial are problematic in their analysis, while the results would possibly be different if statistical and metrological issues had been considered, as already mentioned. Furthermore, the recently published SUPERNOVA study [33], which assesses the feasibility and safety of ECCO_2_R to facilitate ultraprotective ventilation in patients with moderate ARDS, was also included in our review.

Our study has important limitations. First, we observed a high heterogeneity in our results, which was expected considering the changes in critical care practices, differences in design, inclusion criteria, and ECLS technologies over time. Our purpose, however, was to incorporate the entire body of evidence. Second, quantitative results are drawn from a limited number of studies, almost half of all the considered ones in the review, which does not allow for confidence in the consistency of the results. Third, ECLS technologies change over time, but we could not specify a cutoff point to study more biocompatible or modern ECLS techniques. To address temporal changes in critical care and ECLS practices, we considered subgroup analyses including high-quality ECMO studies and LPV strategies, while ECCO_2_R studies were analyzed separately. Furthermore, although we included a number of observational studies, which are more subject to bias than RCTs, we limited our meta-analysis to studies with a higher methodological quality. Finally, we did not include in any meta-analysis subgroup the old RCT by Zapol et al. [17] in which only VA-ECMO was used together with outdated ECMO machines; that study suffered from major limitations, and severe bleeding complications were mentioned too.

One international study is currently evaluating the ECCO_2_R technique, enabling ultra-LPV in ARDS patients. In the UK, the REST clinical trial (Clinical Trials.gov NCT02654327) is evaluating ARDS patients treated with lower tidal volume ventilation versus standard care and is including 1.120 patients with PaO_2_/FiO_2_ <150 mmHg.

## 7. Conclusions

According to our results, ECLS use was not associated with a benefit in mortality rate in patients with ARDS. However, when restricted to higher quality studies, ECMO was associated with a significant benefit in mortality rate. Furthermore, in patients with H1N1, a potential benefit of ECLS was apparent. The current study highlights the significant heterogeneity among the studies and the limited number of high-quality data.

Despite the recent publication of the EOLIA trial, we still urgently need adequately designed RCTs that will allow for the least-biased evaluation of ECLS effectiveness. Until more high-quality data can be derived, ECLS should be left as a salvage therapy option for severe hypoxemic ARDS patients.

## Figures and Tables

**Table 1:** Clinical trials of extracorporeal life support.

	Clinical trial	Study design	Lung protective ventilation in the ECLS group	Lung protective ventilation in the control group	Risk of bias	Outcome
Randomized clinical trials	Zapol et al. [17]	90 pts with acute hypoxemic respiratory failure treated with IMV vs. IMV with VA-ECMO	No	No	Low	No improvement with ECMO use, >90% overall mortality
	Morris et al. [32]	40 pts with acute hypoxemic respiratory failure treated with IRV IMV vs. IMV with VV-ECCO_2_R	No	No	Low	No significant difference in mortality, 67% mortality in the ECCO_2_R group
	Peek et al. CESAR trial [10]	180 pts with ARDS treated with IMV vs. IMV with VV-ECMO	Yes	Yes	Low	Significantly reduced mortality (37% vs. 53% in the controls) and disability in the ECMO group
	Bein et al. Xtravent trial [18]	79 pts with ARDS treated with ultraprotective lung ventilation by ECCO_2_R use vs. conventional IMV	Yes	Yes	Low	Favours ECCO_2_R vs. conventional IMV. More ventilator-free days in pts with P/F ratio <150
	Combes et al. EOLIA trial [9]	249 pts with severe ARDS treated with IMV vs. IMV with VV-ECMO	Yes	Yes	Low	No significant difference in 60-day mortality between the 2 groups

Observational clinical trials	Lewandowski et al. [16]	Cohort prospective observational study: 73 severe ARDS pts treated with ECMO vs. 49 pts treated with IMV	NA	Yes	High	ECMO was not associated with a lower mortality rate
	Mols et al. [15]	Cohort prospective observational study: 245 severe ARDS pts, of which 62 treated with ECMO	Yes	Yes	High	ECMO was associated with a lower mortality rate
	Beiderlinden et al. [14]	Cohort prospective observational study: 150 severe ARDS pts, of which 32 pts treated with ECMO vs. 118 treated with IMV	Yes	N/A	High	Mortality in ECMO pts tended to be higher than that in pts with conservative treatment
	Noah et al. [11]	Cohort prospective observational study: 75 matched pairs of pts with ARDS due to influenza A (H1N1), treated with VV-ECMO vs. no ECMO therapy	Yes	N/A	Low	ECMO use was associated with lower mortality compared with matched non-ECMO-referred patients
	ANZ ECMO Influenza Investigators [29]	Retrospective observational study: influenza A (H1N1) ARDS pts, of which 61 were treated with ECMO vs. 133 without ECMO	N/A	N/A	High	ECMO use was associated with higher mortality compared to conservative treatment
	Roch et al. [13]	Prospective observational study: 18 influenza A (H1N1) ARDS pts, of which 9 were treated with ECMO vs. no ECMO therapy	Yes	Yes	High	ECMO may be an effective salvage treatment in ARDS pts
	Patroniti et al. [30]	Retrospective observational study: 153 influenza A (H1N1) ARDS pts, of which 60 were treated with ECMO vs. no ECMO therapy	N/A	N/A	High	ECMO may be an effective salvage treatment in ARDS pts
	Pham et al. [12]	Prospective observational matched case-control study: pts with ARDS due to influenza A (H1N1), treated with VV-ECMO vs. no ECMO therapy	Yes	Yes	Low	ECMO use was associated with no significant difference in mortality compared with matched non-ECMO-referred patients
	Tsai et al. [28]	Retrospective observational matched case-control study: pts with ARDS, treated with ECMO vs. no ECMO therapy	N/A	Yes	High	ECMO use was associated with lower mortality risk
	Kanji et al. [27]	Retrospective observational cohort matched and unmatched study: pts with severe hypoxemic respiratory failure, treated with ECMO vs. no ECMO therapy	N/A	N/A	High	ECMO use was associated with lower mortality risk but longer ICU and hospital length of stay
	Combes et al. SUPERNOVA trial [33]	Prospective single-arm, phase 2 study: 78 of 95 pts received ultraprotective ventilation	Yes	No control group	—	ECCO_2_R use minimized respiratory acidosis. Relatively high levels of adverse events

Upcoming clinical trial	REST clinical trial (NCT02654327)	RCT: 1120 pts with acute respiratory failure, with PaO_2_/FiO_2_ <150 mmHg	—	—	—	IMV vs. IMV plus ECCO_2_R

RCT: randomized controlled trial; IMV: invasive mechanical ventilation; VA: venoarterial; ECMO: extracorporeal membrane oxygenation; IRV: inverse ratio ventilation; VV: venovenous; ECCO_2_R: extracorporeal carbon dioxide removal; VT: tidal volume; ARDS: acute respiratory distress syndrome; N/A: not applicable; pts: patients; P/F ratio: PaO_2_/FiO_2_ ratio.
